# circGFRA1 and GFRA1 act as ceRNAs in triple negative breast cancer by regulating miR-34a

**DOI:** 10.1186/s13046-017-0614-1

**Published:** 2017-10-16

**Authors:** Rongfang He, Peng Liu, Xiaoming Xie, Yujuan Zhou, Qianjin Liao, Wei Xiong, Xiaoling Li, Guiyuan Li, Zhaoyang Zeng, Hailin Tang

**Affiliations:** 1 The Key Laboratory of Carcinogenesis of the Chinese Ministry of Health, Xiangya Hospital, Central South University, Changsha, Hunan China; 2 The Key Laboratory of Carcinogenesis and Cancer Invasion of the Chinese Ministry of Education, Cancer Research Institute, Central South University, Changsha, Hunan China; 3 Department of Pathology, The First Affiliated Hospital of University of South China, Hengyang, Hunan Province China; 4 Department of Breast Oncology, Sun Yat-sen University Cancer Center; State Key Laboratory of Oncology in South China; Collaborative Innovation Center of Cancer Medicine, Guangzhou, China; 5 Hunan Key Laboratory of Translational Radiation Oncology, Hunan Cancer Hospital and the Affiliated Cancer Hospital of Xiangya School of Medicine, Central South University, Changsha, Hunan China

**Keywords:** Circular RNAs, miR-34a, GFRA1, Competitive endogenous RNAs, Triple negative breast cancer

## Abstract

**Backgroud:**

Accumulating evidences indicate that circular RNAs (circRNAs), a class of non-coding RNAs, play important roles in tumorigenesis. However, the function of circRNAs in triple negative breast cancer (TNBC) is largely unknown.

**Methods:**

We performed circRNA microarrays to identify circRNAs that are aberrantly expressed in TNBC cell lines. Expression levels of a significantly upregulated circRNA, circGFRA1, was detected by quantitative real-time PCR (qRT-PCR) in TNBC cell lines and tissues. Kaplan-Meier survival analysis was used to explore the significance of circGFRA1 in clinical prognosis. Then, we examined the functions of circGFRA1 in TNBC by cell proliferation, apoptosis and mouse xenograft assay. In addition, luciferase assay was used to explore the miRNA sponge function of circGFRA1 in TNBC.

**Results:**

Microarray analysis and qRT-PCR verified a circRNA termed circGFRA1 that was upregulated in TNBC. Kaplan-Meier survival analysis showed that upregulated circGFRA1 was correlated with poorer survival. Knockdown of circGFRA1 inhibited proliferation and promoted apoptosis in TNBC. Via luciferase reporter assays, circGFRA1 and GFRA1 was observed to directly bind to miR-34a. Subsequent experiments showed that circGFRA1 and GFRA1 regulated the expression of each other by sponging miR-34a.

**Conclusions:**

Taken together, we conclude that circGFRA1 may function as a competing endogenous RNA (ceRNA) to regulate GFRA1 expression through sponging miR-34a to exert regulatory functions in TNBC. circGFRA1 may be a diagnostic biomarker and potential target for TNBC therapy.

## Background

Breast cancer is the most commonly diagnosed cancer in women worldwide. It is estimated that there will be 255,180 new cases and 41,070 deaths of breast cancer in the United States in 2017 [1]. The last few decades have witnessed outstanding advances in breast cancer treatment. However, the prognosis for triple negative breast cancer (TNBC) remains poor. Therefore, it is significant to develop more effective therapeutic strategies to treat breast cancer, especially TNBC.

Circular RNAs (circRNAs) are a class of non-coding RNAs that are widely expressed in mammals [2]. A plenty of circRNAs have been identified, but their potential functions are poorly understood. There are currently few reports describing the role of circRNAs in breast cancer. Liang G et al. reported that circDENND4C is a HIF1α-associated circRNA promoting the proliferation of breast cancer under hypoxia [3]. Lu L et al. provided a profile of circRNAs in breast cancer and adjacent normal-appearing tissues [4]. However, the function of circRNAs in TNBC progression is unclear. Revealing the role of circRNAs will be critical for understanding TNBC pathogenesis and offering a novel insight into identificating new biomarkers or therapeutic targets of TNBC.

microRNAs (miRNAs) are endogenous, non-protein-coding, single-stranded 19- to 25-nucleotide RNAs that play a vital role in the process of cancer [5]. miR-34a has been reported to act as a tumor suppressor to regulate tumor progression and is always down-regulated in cancers [6], including prostate cancer [7], glioblastoma [8], colon cancer [9] and breast cancer [10–12]. Due to the significant role that miR-34a plays in cancer, development of miR-34a-based gene therapy is encouraged for multiple types of cancers.

It is reported that RNAs can act as competitive endogenous RNAs (ceRNAs) to co-regulate each other by competing for shared microRNAs [13, 14]. Studies by several groups have illustrated that mRNAs, pseudogenes, long noncoding RNAs (lncRNAs) and circRNAs may all serve as ceRNAs [15]. circRNAs in mammals have also been shown to function as miRNA sponges or ceRNAs. Memczak et al. found that the circRNA CDR1as binds to miR-7 and impairs midbrain development [16]. Hansen TB et al. showed that the circRNA Sry functions as a miR-138 sponge [17]. Zhong Z et al. found that circRNA-MYLK might function as ceRNA for miR-29a, which could contribute to EMT and the development of bladder cancer through activating VEGFA/VEGFR2 and downstream Ras/ERK signaling pathway [18]. All these findings indicate that circRNAs could function as miRNA sponges to contribute to the regulation of cancers.

In this study, we analyzed the expression profiles of circRNAs in TNBC cell lines through microarrays. Expression levels of a significantly upregulated circRNA, circGFRA1, was detected by quantitative real-time PCR (qRT-PCR) in TNBC cell lines and tissues. Kaplan-Meier survival analysis showed that upregulated circGFRA1 was correlated with poorer survival in TNBC. We examined the functions of circGFRA1 in TNBC and found that knockdown of circGFRA1 could inhibit cell proliferation and induce apoptosis. In addition, luciferase assay showed that circGFRA1 could bind to miR-34a. Furthermore, GFRA1 was also a direct target of miR-34a. Taken together, we conclude that circGFRA1 may act as a ceRNA to regulate GFRA1 expression by decoying miR-34a, indicating that circGFRA1 can be used as a diagnostic biomarker and potential target in TNBC therapy.

## Methods

### Patients samples

Tumor and paired adjacent normal mammal tissues from TNBC patients who received treatment at Sun Yat-Sen University Cancer Center were collected and immediately cut and stored in RNAlater (Ambion) and subjected to quantitative real-time PCR (qRT-PCR) analysis. None of these patients received neoadjuvant therapy. This study was approved by the Ethics Committee of Sun Yat-Sen University Cancer Centre Health Authority and in accordance with the ethical standards formulated in the Declaration of Helsinki. All participants provided written informed consent.

### Cell lines and culture

All the cell lines were obtained from American Type Culture Collection (Manassas, USA), including human mammary epithelial (HME) cell lines (MCF10A and 184A1) and breast cancer cell lines (SKBR3, T47D, BT474, MCF-7, BT-483, BT-20, BT549, MDA-MB-468 and MDA-MB-231). All the cell lines were passaged in our laboratory for less than six months and maintained according to the supplier’s instructions. The cell lines were found to be free of mycoplasma infection and authenticity verified by DNA fingerprinting before use.

### Microarray analysis

Three TNBC cell lines (MDA-MB-231, BT549 and MDA-MB-468) and normal mammary epithelial cell line (MCF-10A) were analyzed by Arraystar Human circRNA Array V2. Total RNA was quantified using NanoDrop ND-1000. The sample preparation and microarray hybridization were performed based on the Arraystar’s standard protocols. Briefly, total RNAs were digested with Rnase R (Epicentre Technologies, USA) to remove linear RNAs and enrich circular RNAs. Then, the enriched circRNAs were amplified and transcribed into fluorescent cRNA utilizing a random priming method (Arraystar Super RNA Labeling Kit). The labeled cRNAs were hybridized onto the Arraystar Human circRNA Array V2 (8x15K, Arraystar). After washing the slides, the arrays were scanned by the Agilent Scanner G2505C. Agilent Feature Extraction software (version 11.0.1.1) was used to analyze the acquired array images. Quantile normalization and subsequent data processing was performed using the R software limma package. Differentially expressed circRNAs were identified through Fold Change filtering. Hierarchical Clustering was performed to show the distinguishable circRNAs expression pattern among samples.

### Quantitative real-time PCR (qRT-PCR)

Total RNA was isolated using TRIzol reagent (Life Technologies, USA). The nuclear and cytoplasmic fractions were isolated using NE-PER Nuclear and Cytoplasmic Extraction Reagents (Thermo Scientific). Complementary DNA was synthesized using the PrimeScript RT reagent kit (Takara Bio Inc., China), and RT-PCR was performed using SYBR Premix Ex Taq (Takara Bio Inc.). The primers for circGFRA1 are F: 5′-CCTCCGGGTTAAGAACAAGC-3′, R: 5′-CTGGCTGGCAGTTGGTAAAA-3′. The primers for GFRA1 are F: 5′-CCAAAGGGAACAACTGCCTG-3′, R: 5′-CGGTTGCAGACATCGTTGGA-3′. The threshold cycle (CT) value for circGFRA1 or GFRA1 was normalized against the CT value for control β-actin, while U6 snRNA was used as an internal control for the relative amount of miR-34a. The relative fold-change in expression with respect to a control sample was calculated by the 2-ΔΔCt method. All the real-time PCR assays were performed with the Bio-Rad IQTM5 Multicolour Real-Time PCR Detection System (USA).

### CCK8 assay

Cell proliferation was assessed by Cell Counting Kit-8 assay (Dojindo Laboratories, Japan). Cells (1 × 10^3^) were seeded into 96-well plates and incubated at 37 °C for 24 h before transfection. CCK-8 solution(10 μl) was added to each well 48 h after transfection. After 2 h of incubation at 37 °C, the absorbance at 450 nM was measured using Spectra Max 250 spectrophotometer (Molecular Devices, USA). Triplicate independent experiments were performed.

### Colony formation assay

Six-well plates were covered with a layer of 0.6% agar in medium supplemented with 20% fetal bovine serum. A total of 1000 cells were prepared in 0.3% agar and cultured for 2 weeks at 37 °C. The numbers of colonies per well were fixed and stained with crystal violet, imaged and counted. Triplicate independent experiments were performed.

### Cell apoptosis assay

For apoptosis assay, cells were stained by propidium iodine/Annexin V-FITC staining (BD Biosciences) then analyzed by flow cytometry FACS Calibur instrument (BD Biosciences) according to the manufacturer’s instructions.

### Mouse xenograft model

All animal studies were approved by the Institutional Animal Care and Use Committee (IACUC) of Sun Yat-Sen University Cancer Center. Standard animal care and laboratory guidelines were followed according to the IACUC protocol. 4-week old female BALB/c nude mice were injected subcutaneously with 2 × 10^6^ cancer cells (5 mice per group) and treated with intratumoral injection (40 μL si-NC or si-circGFRA1). Tumor volumes were measured every 4 days for 28 days and calculated by the formula: volume = length × (width/2)^2^.

### Nuclear-cytoplasmic fractionation

Cytoplasmic and nuclear RNA Isolation were performed with PARIS™ Kit (Invitrogen, USA) following the manufacturer’s instruction. Briefly, the cells were lysed with cell fractionation buffer and centrifuged to separate the nuclear and cytoplasmic cell fractions. The supernatant was transferred to a fresh RNase-free tube. The remaining lysate was washed with cell fractionation buffer and centrifuged. Cell disruption buffer was added to lyse the nuclei. Mix the lysate and the supernatant above with a 2× lysis/binding solution and add equal volume of ethanol Draw the mixture through a filter cartridge then wash the sample with wash solution. The RNA of cytoplasmic and nuclear was eluted with elution solution.

### Luciferase reporter assay

Luciferase reporter vector with the full length of the 3′-UTR of circGFRA1 or GFRA1 and the mutant version were constructed. Luciferase reporter vector with miR-34a mimics or miR-34a inhibitors was transfected into MDA-MB-231 cells. After 48 h of incubation, the firefly and Renilla luciferase activities were quantified with a dual-luciferase reporter assay (Promega, USA).

### RNA immunoprecipitation (RIP) assay

The MS2bp-MS2bs based RIP assay was performed as follows. Briefly, cells were co-transfected with MS2bs-circGFRA1 vector, MS2bs-circGFRA1mt vector (the miR-34a complementary sites in circGFRA1 was mutated by deletions to remove complementarity to miR-34a) or blank control vector MS2bs-Rluc together with MS2bp-GFP using Lipofectamine 2000 (Invitrogen, USA). After 48 h, cells were used to perform RIP using a GFP antibody (Roach, USA) and the Magna RIP RNA-Binding Protein Immunoprecipitation Kit (Millipore, USA) according to the manufacturer’s instructions. The complexes of RNA were then treated with Trizol (Life Technologies, USA) for further purification and the miR-34a level was analyzed by qRT-PCR.

### Western blot analysis

Western blot analysis was performed using standard procedures. Briefly, total proteins were extracted and separated by 10% sodium dodecyl sulfate polyacrylamide gel electrophoresis (SDS-PAGE) and transferred onto a polyvinylidene difluoride (PVDF) membrane (Millipore, USA). To block nonspecific binding, the membranes were incubated with 5% skim milk powder at room temperature for one hour. The membrane was then incubated with primary antibody against GFRA1 (1:1000, CST, USA), followed by horseradish peroxidase (HRP)-labeled secondary antibody (Santa Cruz) and detected by chemiluminescence. An anti-β-actin antibody (1:1000, Affinity, USA) was used as a protein loading control.

### Statistical analysis

Comparisons between groups were analyzed with t tests and χ^2^ tests. Overall survival (OS) and disease-free survival (DFS) curves were plotted using the Kaplan-Meier method and survival differences were evaluated using a log-rank test. Pairwise expression correlation was analyzed by Pearson correlation tests. *P* < 0.05 was considered as statistically significant. The statistical analysis was performed using SPSS 19.0 software.

## Results

### circRNA expression profiles in TNBC

**Fig. 1:**
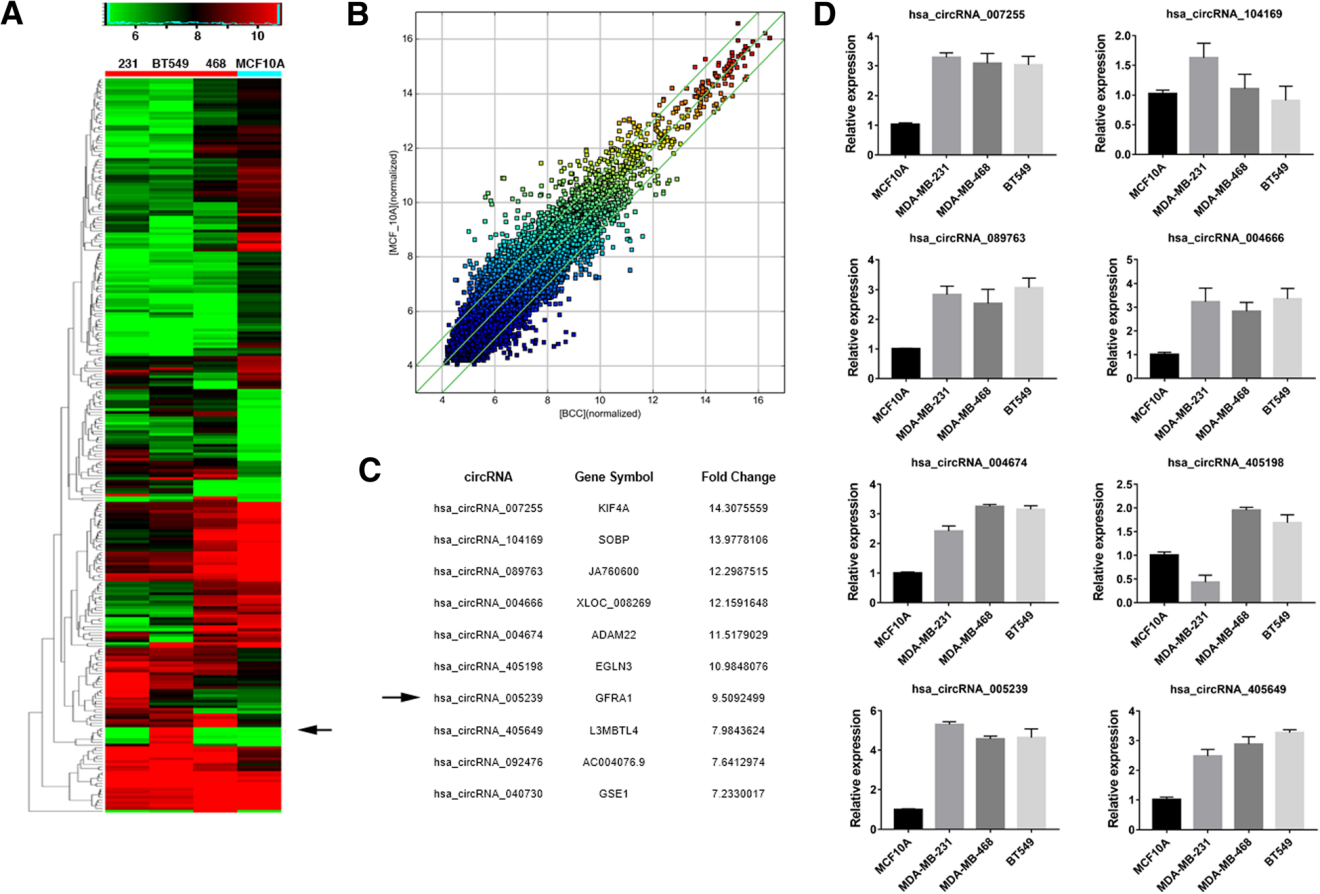
circRNA expression profiles in TNBC. **a.** The cluster heat map showed the differentially expressed circRNAs over 3-fold change. Red color indicates high expression level, and green color indicates low expression level. The black arrow indicates circGFRA1. **b.** The scatter plot was used for assessing the variation in circRNA expression between TNBC cell lines and HME cell line. The values of x and y axes in the scatter plot were the normalized signal values of the samples (log2 scaled). The green lines are fold-change lines. **c.** The top 10 upregulated circRNAs were shown. **d.** qRT-PCR was performed to verify the expression of the top eight upregulated circRNAs. All the data are shown as the mean ± s.e.m.

To investigate the potential involvement of circRNAs in TNBC, we performed high throughput circRNAs microarray assay on three TNBC cell lines (MDA-MB-231, MDA-MB-468 and BT549) and one HME cell line (MCF-10A). Hierarchical clustering showed the circRNAs expression pattern (Fig. 1a) and the variation of circRNAs expression was revealed in the scatter plots (Fig. 1b). The result showed that 174 circRNAs were downregulated and 93 circRNAs were upregulated with fold change greater than 3, *P* < 0.05 and FDR < 0.05.

The top 10 upregulated circRNAs were listed in Fig. 1c and qRT-PCR was performed to verify the expression of the top eight upregulated circRNAs (Fig. 1d). The expression of hsa_circ_005239 was the top upregulated circRNA in TNBC cell lines. According to human reference genome (GRCh37/hg19), we further assumed that hsa_circ_005239, located at chr10:117,849,251–117,856,275, is derived from gene GFRA1 (GDNF family receptor alpha 1), which is located on chromosome 10q25. Thus, we termed hsa_circ_005239 as “circGFRA1”.

### circGFRA1 is upregulated and correlated with poor clinical outcomes in TNBC

**Fig. 2:**
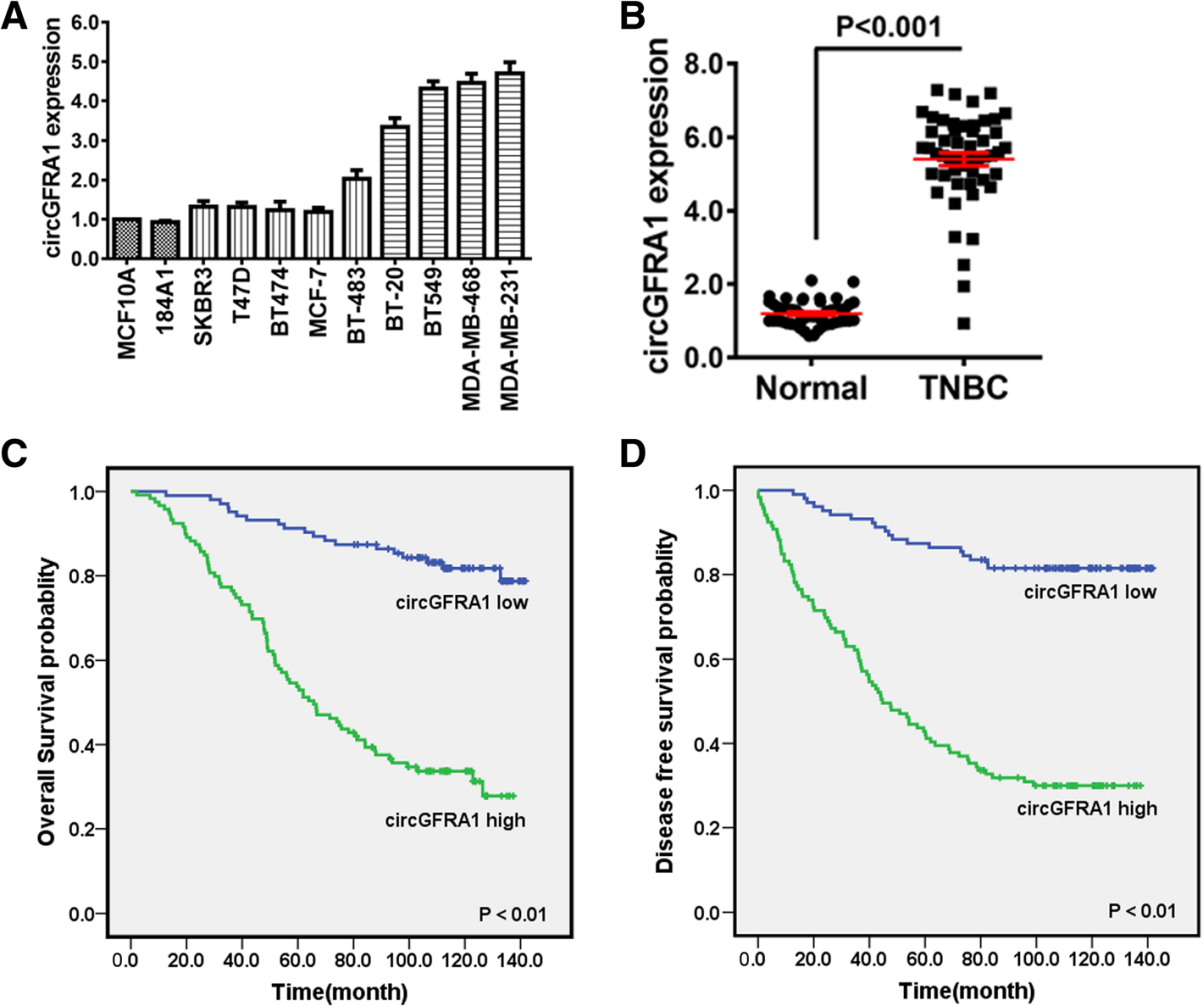
circGFRA1 is upregulated and correlated with poor clinical outcomes in TNBC. **a.** The expression level of circGFRA1 was determined by qRT-PCR in 11 different mammary cell lines, including two HME cell line (MCF-10A and 184A1) and nine breast cancer cell lines. circGFRA1 expression was normalized using β-actin expression. **b.** The expression level of circGFRA1 in 51 TNBC tissues and their matched normal adjacent tissues was determined by qRT-PCR. **c.** OS curves for 222 TNBC patients with high or low circGFRA1 expression. **d.** DFS curves for 222 TNBC patients with high or low circGFRA1 expression. All the data are shown as the mean ± s.e.m.

We further confirmed the expression level of circGFRA1 in 11 breast cell lines, including nine breast cancer cell lines and two HME cell lines. As Fig. 2a showed, circGFRA1 was up-regulated especially in TNBC cell lines (including BT-20, BT549, MDA-MB-468 and MDA-MB-231). Moreover, the expression level of circGFRA1 was evaluated in 51 TNBC tissues and their paired adjacent normal tissues (Fig. 2b). The results showed that circGFRA1 was significantly up-regulated in TNBC tissues.

Next, we explored the potential clinicopathological implications of circGFRA1 in TNBC. The tissues of 222 TNBC patients were used for qRT-PCR analysis of circGFRA1 expression. Patients were divided into high or low expression groups based on circGFRA1 expression level. Patients with circGFRA1 expression levels equal to or greater than the average circGFRA1 expression level were defined as “circGFRA1 high” group. We found that circGFRA1 expression level was positively correlated with tumor size, TNM staging, lymph node metastasis and histological grade but not with age or menopause (Table 1). These results revealed that circGFRA1 might play a vital role in tumor progression of TNBC.

**Table 1:** Clinicopathological variables and circGFRA1 expression in 222 breast cancer patients

Variables	Cases (*n* = 222)	circGFRA1				*P* value
		Low		High		
Age (years)						0.216
≤ 50	148	73	49.3%	75	50.7%	
> 50	74	30	40.5%	44	59.5%	
menopause						0.738
no	131	62	47.3%	69	52.7%	
yes	91	41	45.1%	50	54.9%	
Tumor size (cm)						0.029*
≤ 2	70	40	57.1%	30	42.9%	
> 2	152	63	41.4%	89	58.6%	
TNM Staging						<0.001*
I-II	155	93	60.0%	62	40.0%	
III-IV	67	10	14.9%	57	85.1%	
LN Infiltrated						<0.001*
No	117	87	74.4%	30	25.6%	
Yes	105	16	15.2%	89	84.8%	
Histological grade						0.036*
Well differentiated	2	2	100.0%	0	0.0%	
Moderately differentiated	109	57	52.3%	52	47.7%	
Poorly differentiated	111	44	39.6%	67	60.4%	

*P < 0.05, statistically significant

To explore the significance of circGFRA1 in clinical prognosis, we used Kaplan-Meier survival analysis to make the OS and DFS curves. The results showed that patients with high circGFRA1 expression level had shorter OS and DFS than patients with low circGFRA1 expression level (Fig. 2c and d). These results demonstrated that high circGFRA1 expression level was significantly associated with poor TNBC outcomes.

### Knockdown of circGFRA1 inhibits proliferation and promotes apoptosis in TNBC

**Fig. 3:**
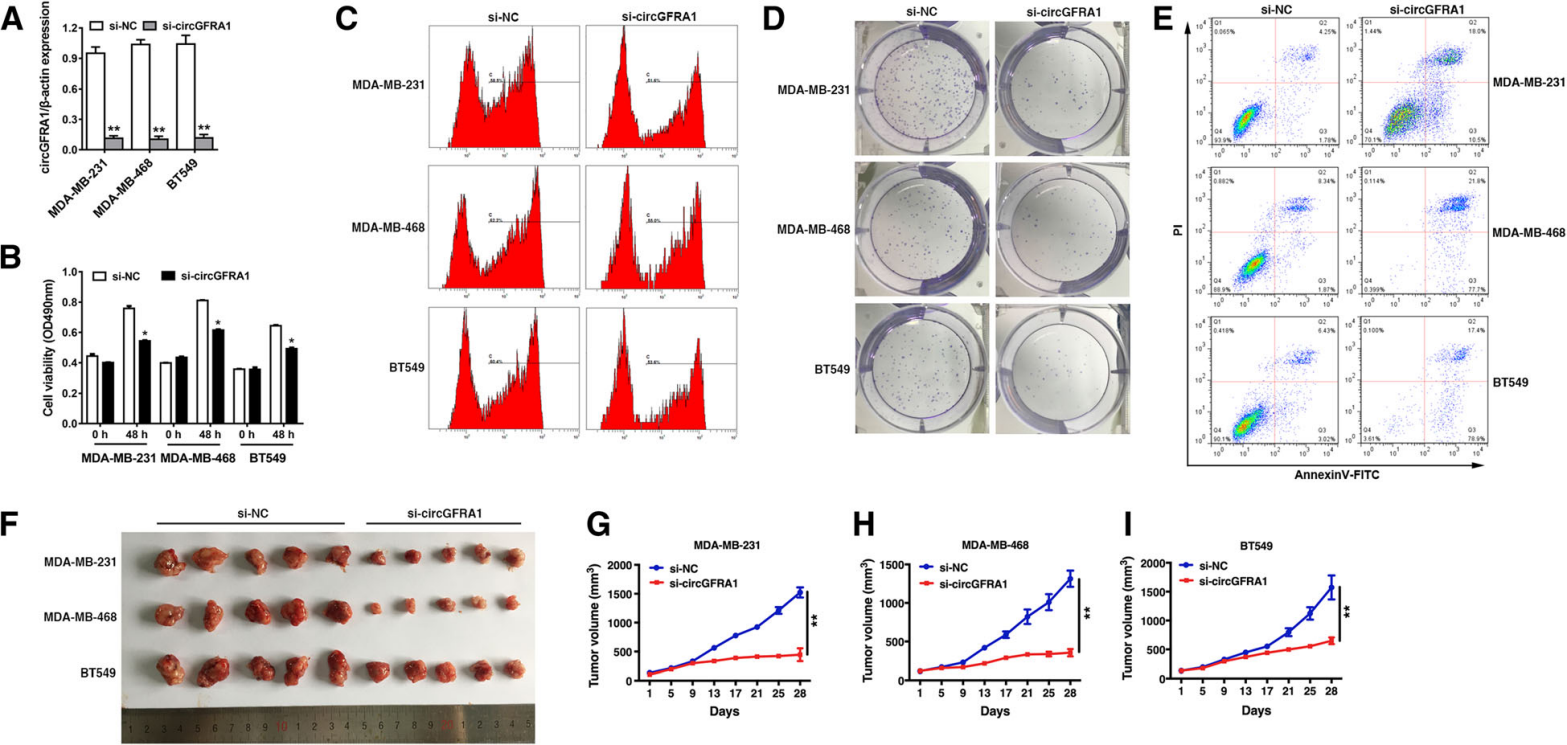
Knockdown of circGFRA1 inhibits proliferation and promotes apoptosis in TNBC. **a.** Cells were transfected with si-NC or si-circGFRA1, qRT-PCR analysis demonstrated that the transfection was successful. **b.** CCK8 assay was performed to assess cell proliferation. **c.** EdU assay was performed to assess cell proliferation. **d.** Colony formation assay was performed to assess cell colony forming ability. **e.** Apoptosis assay was performed after transfection. **f.** In vivo xenograft models were performed (5 mice per group). **g-i.** Tumor volume was monitored every 4 days for 28 days. All the data are shown as the mean ± s.e.m., *P < 0.05 and **P < 0.01

Next, we used RNA interference to knock down the expression of circGFRA1 to evaluate its biological functions in TNBC. qRT-PCR analysis demonstrated that the inhibition was successful (Fig. 3a). Subsequent cell proliferation assay revealed that downregulation of circGFRA1 significantly suppressed the growth of TNBC cells (Fig. 3b). Furthermore, 5-ethynyl-20 -deoxyuridine (EdU) assay showed that the proliferation potential of TNBC cells was impaired upon downregulation of circGFRA1 (Fig. 3c). In colony formation assay, colony-forming ability of TNBC cells were significantly reduced after downregulation of circGFRA1 (Fig. 3d). Apoptosis assay showed that downregulation of circGFRA1 markedly promoted the apoptosis of TNBC cells (Fig. 3e). To further investigate the biological significance of circGFRA1 in tumor growth in vivo, xenograft experiments were performed. And we found that inhibition of circGFRA1 led to a significant decrease in tumor growth (Fig. 3f-3i). All these findings suggest that circGFRA1 could promote proliferation and inhibit apoptosis in TNBC.

### circGFRA1 serves as a sponge for miR-34a

**Fig. 4:**
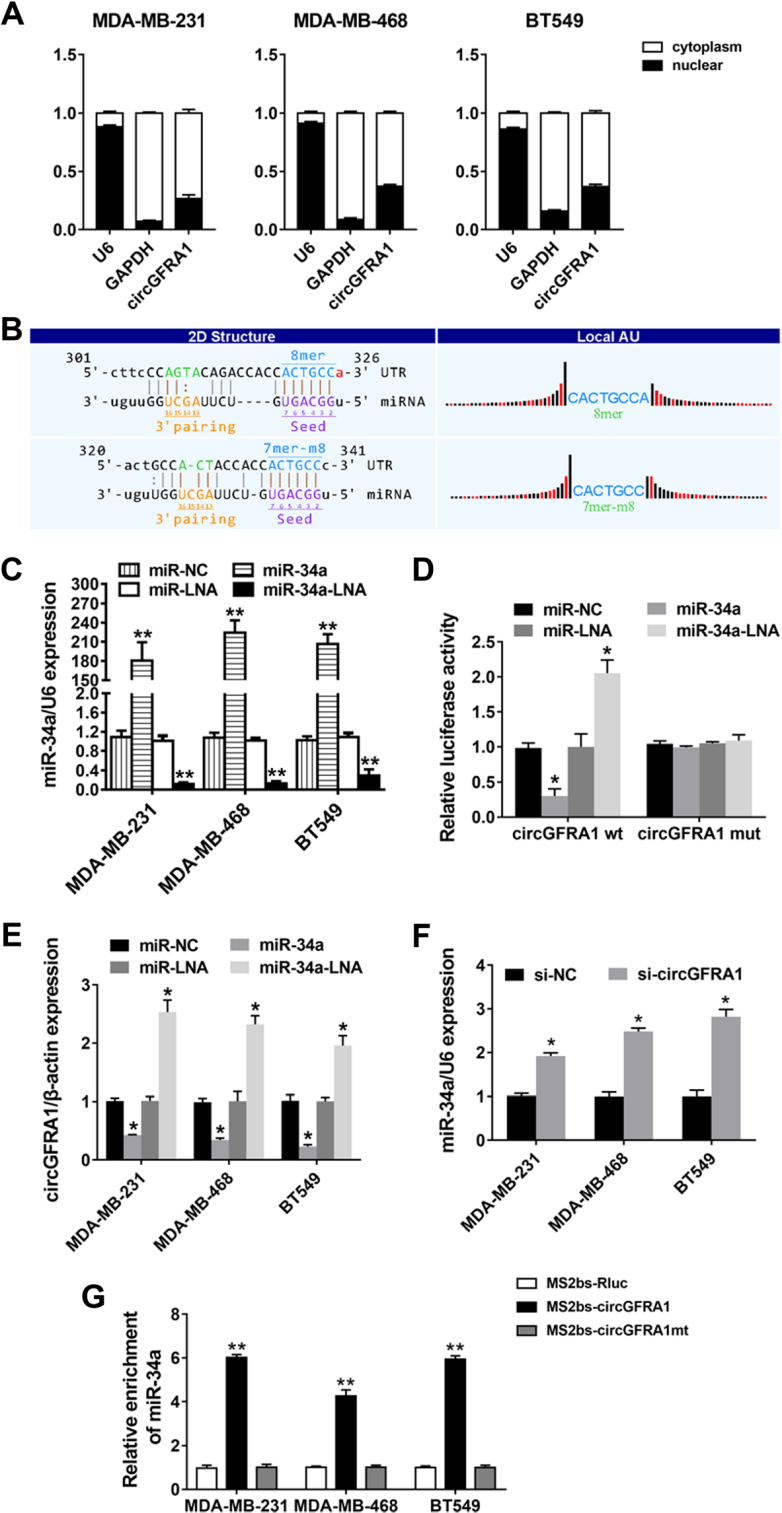
circGFRA1 serves as a sponge for miR-34a. **a.** The levels of nuclear control transcript (U6), cytoplasmic control transcript (GAPDH mRNA) and circGFRA1 were assessed by qRT-PCR in nuclear and cytoplasmic fractions. **b.** The predicted binding sites of miR-34a within circGFRA1 were shown. **c.** Cells were transfected with miR-34a mimic or miR-34a inhibitor, qRT-PCR analysis demonstrated that the transfection was successful. **d.** Luciferase assay of MDA-MB-231 cells cotransfected with miR-34a mimics or miR-34a inhibitor and luciferase reporter containing circGFRA1 3′-UTR (circGFRA1 wt) or mutant construct (circGFRA1 mut). **e.** Cells were transfected as described, and the expression of circGFRA1 was determined by qRT-PCR. **f.** Cells were transfected with si-NC or si-circGFRA1, and the expression of miR-34a was determined by qRT-PCR. U6 snRNA was used as an internal control. **g.** MS2-based RIP assay in TNBC cells transfected with MS2bs-circGFRA1, MS2bs-circGFRA1mt, or MS2bs-Rluc (control vector) (*n* = 3). All the data are shown as the mean ± s.e.m., *P < 0.05 and **P < 0.01

Next, we characterized the intracellular location of circGFRA1 in TNBC cell lines. Nuclear and cytoplasmic fractions were separated from cells and the levels of the nuclear control transcript (U6) and cytoplasmic control transcript (GAPDH mRNA) were detected by qRT-PCR, respectively. The results revealed that circGFRA1 mostly distributed in the cytoplasm of TNBC cells (Fig. 4a). As circGFRA1 was predominantly localized in the cytoplasm, it might function as a ceRNA to sequester miRNAs, leading to the liberation of corresponding miRNA-targeted transcripts. Subsequently, we explored whether circGFRA1 could act as a miRNA sponge. The circRNA/miRNA interaction was predicted with Arraystars’s home-made miRNA target prediction software based on TargetScan and miRanda. Potential binding sites of miR-34a were found within the circGFRA1 sequence (Fig. 4b). Thus, we performed luciferase reporter assays to determine whether miR-34a could directly target the 3′UTR of circGFRA1. qRT-PCR analysis demonstrated that the transfection was successful (Fig. 4c). The luciferase intensity was reduced by more than 40% when cells were co-transfection with luciferase reporters and miR-34a mimics (Fig. 4d). Moreover, the luciferase intensity increased when cells were co-transfection with luciferase reporters and miR-34a LNA (Fig. 4d). To confirm the direct interaction between miR-34a and circGFRA1, the MREs of miR-34a in the luciferase reporter were mutated. We found that co-transfection of the mutated luciferase reporter and miR-34a mimics or miR-34a LNA had no significant effect on luciferase activity (Fig. 4d). qRT-PCR analysis further confirmed that miR-34a mimics could inhibit the expression of circGFRA1 while miR-34a LNA increased circGFRA1 expression (Fig. 4e). And the expression of miR-34a was upregulated after inhibition of circGFRA1 (Fig. 4F). To validate the direct binding between circGFRA1 and miR-34a, we performed RIP assay with MS2-binding protein (MS2bp), which specifically binds RNA containing MS2-binding sequences (MS2bs), to pull down endogenous miRNAs associated with circGFRA1. We generated a construct containing circGFRA1 transcript combined with MS2bs elements (named MS2bs-circGFRA1) and cotransfected it into TNBC cells with a construct containing MS2bp-GFP. The immunoprecipitation was then performed using GFP antibody and IgG was used as a negative control. And miR-34a expression was analyzed using qRT-PCR. Figure 4g showed that miR-34a was significantly enriched in RNAs retrieved from MS2bs-circGFRA1 compared with that from control MS2bs-Renilla luciferase (named MS2bs-Rluc) and MRE-mutated MS2bs-circGFRA1 (named MS2bs-circGFRA1mt). No significant difference was shown in miR-34a enrichment between MS2bs-circGFRA1mt group and control MS2bs-Rluc group, indicating the specific interaction between circGFRA1 and miR-34a. In line with luciferase assays, our data confirmed that circGFRA1 functionally interacts with miR-34a and serves as a sponge for miR-34a.

### circGFRA1 and GFRA1 act as ceRNAs in TNBC through regulation of miR-34a

**Fig. 5:**
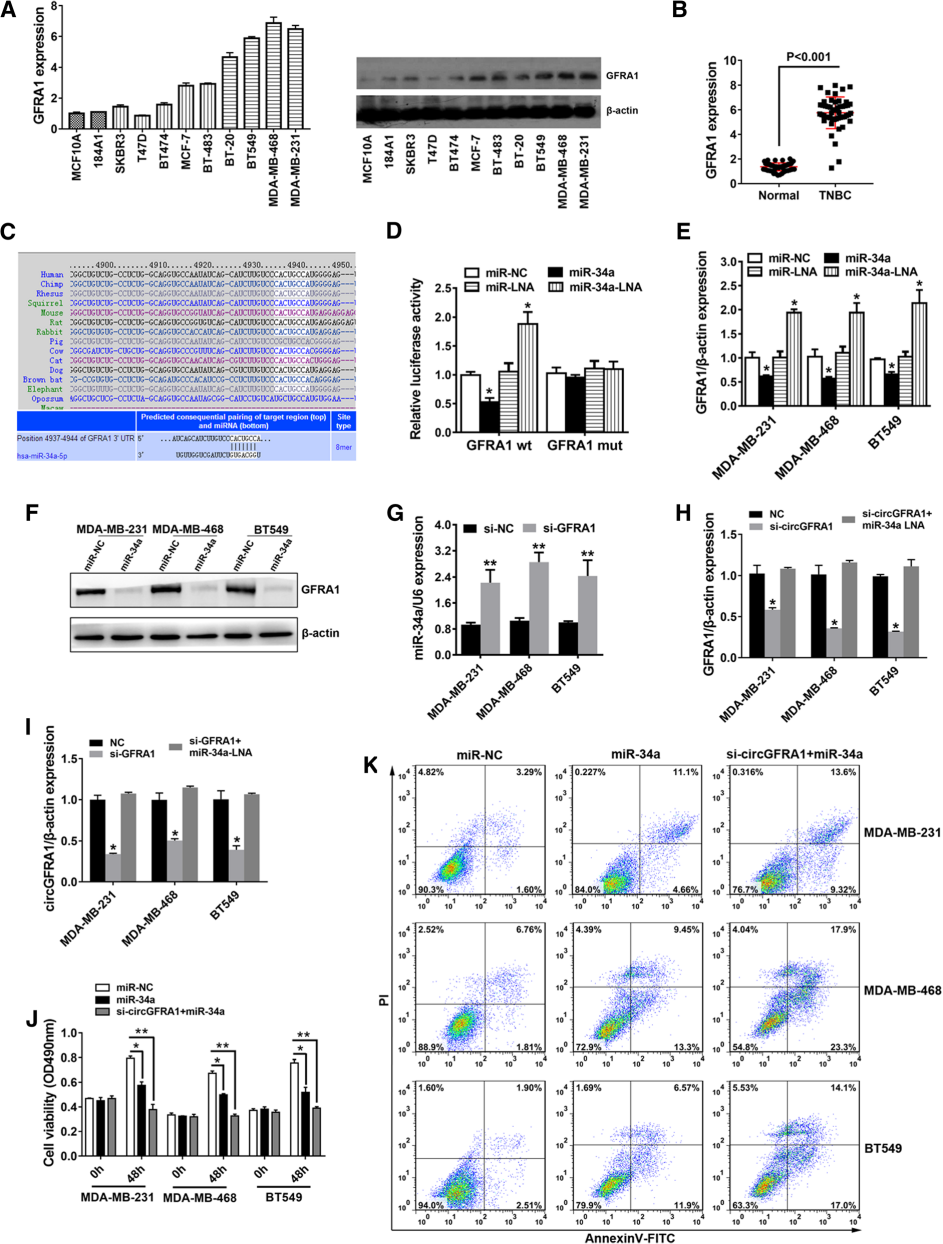
circGFRA1 and GFRA1 act as ceRNAs in TNBC through regulation of miR-34a. **a.** The expression level of GFRA1 was determined by qRT-PCR (left) and western blot (right) in 11 mammary cell lines. β-actin was used as a control. **b.** The expression level of GFRA1 in 51 TNBC tissues and their matched normal adjacent tissues was determined by qRT-PCR. **c.** The predicted binding sites of miR-34a within GFRA1 were shown. **d.** Luciferase assay of MDA-MB-231 cells cotransfected with miR-34a mimics or miR-34a inhibitor and luciferase reporter containing GFRA1 3′-UTR (GFRA1 wt) or mutant construct (GFRA1 mut). **e.** Cells were transfected as described, and the expression of GFRA1 was determined by qRT-PCR. **f.** Cells were transfected as described, and the expression of GFRA1 was determined by western blot analysis. **G.** Cells were transfected with si-NC or si-GFRA1, and the expression of miR-34a was determined by qRT-PCR. **h.** Cells were transfected with si-NC, si-circGFRA1 or si-circGFRA1 + miR-34a inhibitor, and the expression of GFRA1 was determined by qRT-PCR. **i.** Cells were transfected with si-NC, si-GFRA1 or si-GFRA1 + miR-34a inhibitor, and the expression of circGFRA1 was determined by qRT-PCR. **j.** Cells were transfected with miR-NC, miR-34a or si-circGFRA1 + miR-34a, and CCK8 assay was performed to assess cell proliferation. **k.** Cells were transfected as described, and apoptosis assay was performed after transfection. All the data are shown as the mean ± s.e.m., *P < 0.05 and **P < 0.01

To explore whether circGFRA1 acts as a ceRNA to sequester miR-34a and liberate the expression of GFRA1, we continued to detect the expression of GFRA1 in breast cancer cell lines and tissues. The results showed that GFRA1 was also up-regulated in TNBC cell lines (Fig. 5a) and tissues (Fig. 5b). Furthermore, we used TargetScan to identify the putative target genes of miR-34a and GFRA1 was predicted (Fig. 5c). To confirm this finding, we constructed a luciferase reporter vector with the full length of GFRA1 3′-UTR containing target sites for miR-34a and a mutant version. Luciferase reporter vector, with miR-34a mimics, was transfected into MDA-MB-231 cells. A significant decrease of luciferase activity was observed when co-transfected with miR-34a mimics, but not with scrambled oligonucleotide (Fig. 5d). However, there was not a significant difference in luciferase activity when co-transfected with mutant luciferase reporter. On the contrary, a significant increase of luciferase activity was observed when co-transfected with miR-34a inhibitors (Fig. 5d). qRT-PCR and Western blot analyses validated that the expression of GFRA1 was reduced by miR-34a (Fig. 5e and e). Moreover, the expression of miR-34a was upregulated by GFRA1 inhibition (Fig. 5g). These results indicated that GFRA1 is a direct target of miR-34a and could also sequester miR-34a.

Next, we detected the mRNA levels of GFRA1 after knockdown of circGFRA1 and found a repression of GFRA1 (Fig. 5h). To prove whether circGFRA1 functions as a ceRNA, we co-transfected miR-34a inhibitor and si-circGFRA1 into TNBC cell lines, and observed that the repression was reversed (Fig. 5h). We further detected the expression levels of circGFRA1 after knockdown of GFRA1. The result showed a decreased expression of circGFRA1. And it was also reversed through co-transfection with miR-34a inhibitors and si-GFRA1 (Fig. 5i). These results suggest that circGFRA1 and GFRA1 serve as ceRNAs by harboring miR-34a to modulate the progress of TNBC.

Since circGFRA1 could serve as a ceRNA to regulate the progress of TNBC by sequestering miR-34a, we further explored whether miR-34a and knock down of circGFRA1 could inhibit the progress of TNBC synergistically. Subsequent cell proliferation assay and apoptosis assay revealed that miR-34a combined with downregulation of circGFRA1 further suppressed the growth and promoted the apoptosis of TNBC cells (Fig. 5j & 5k).

## Discussion

Many miRNAs and lncRNAs have been reported to regulate TNBC development. However, whether circRNAs play a role in TNBC is unknown. This is the first report on the expression profile and regulatory function of circRNAs in TNBC. In this study, a number of aberrantly expressed circRNAs in TNBC cell lines were identified. We found that circGFRA1 was upregulated and correlated with poor clinical outcomes in TNBC. Moreover, we found that circGFRA1 was positively correlated with tumor size, TNM staging, lymph node metastasis and histological grade of TNBC. Further experiments showed that circGFRA1 could promote proliferation and inhibit apoptosis in TNBC. These results revealed that circGFRA1 plays a vital role in TNBC progression and may be a potential prognostic biomarker and therapeutic target of TNBC.

The ceRNA hypothesis was based on numerous evidences and described how RNAs communicate with each other via competing for binding to miRNAs and regulating the expression of each other to construct a complex posttranscriptional regulatory network [13, 14]. mRNAs, pseudogenes, lncRNAs and circRNAs may all serve as ceRNAs [15]. CD44 3′ UTR overexpressed in breast cancer cells could interact with endogenous miRNAs to arrest their mRNA-targeting function [19]. Pseudogene PTENP1 could regulate cellular levels of PTEN and exert a growth-suppressive role [20]. BRAF pseudogene could act as a ceRNA and elevate BRAF expression and MAPK activation [21]. LncARSR promoted sunitinib resistance by competitively binding miR-34/miR-449 to facilitate AXL and c-MET expression [22]. Linc-RoR functions as a ceRNA to regulate the expression of OCT4, SOX2 and NANOG in embryonic stem cells [23]. In fact, a few circRNAs have also been confirmed as functional miRNA sponges. A circRNA named CDR1as was first reported to function as a sponge of miR-7 [16]. Another circRNA called Sry was reported to serve as a sponge for miR-138 [17]. These findings indicate that circRNAs could function as miRNA sponges to contribute to the regulation of cancers.

miR-34a has been reported to regulate tumor progression in many cancers. In prostate cancer, miR-34a negatively regulates CD44 to inhibit cancer regeneration and metastasis [7]. In glioblastoma, miR-34a is identified as a tumor suppressor due to its regulation of the TGF-β signaling network [8]. In colon cancer, miR-34a suppresses cancer stem cells self-renewal and differentiation by targeting Notch1 [9]. Previously, we found that miR-34a could inhibit the proliferation and migration of breast cancer by targeting B-cell lymphoma 2 (Bcl-2), silent information regulator 1 (SIRT1), E2F transcription factor 3 (E2F3) and CD44 [10, 11]. Moreover, we found that miR-34a targets LDHA to regulate metabolism in breast cancer [12]. Due to the significant role that miR-34a plays in cancer, development of miR-34a-based gene therapy is encouraged for multiple types of cancers.

GFRA1 is a cell surface receptor for glial cell line-derived neutrophic factor (GDNF). GFRA1 is expressed in several human cancers, such as prostate cancer [24] and hepatocellular carcinoma [25], and involved in tumorigenesis through regulation of migration and invasion [26]. In human pancreatic cancers GFRA1 is highly expressed and associated with poorer survival [27]. And methylation status of GFRA1 could be used as potential biomarkers for the screening of rectal cancer [28]. Moreover, GFRA1 could reduce cisplatin-induced cell apoptosis and significantly increased osteosarcoma cell survival via autophagy [29]. It has been reported that GFRA1 is overexpressed in breast cancer [30] and positively associated with lymphovascular invasion, lymph node metastasis and advanced stages [31]. Moreover, GFRα1 expression were significantly associated with survival outcome of breast cancer [32]. Thus, GFRA1 may be useful predictors of disease progression and outcome of breast cancers.

In this study, we found that miR-34a could target both circGFRA1 and GFRA1, suggesting that circGFRA1 might function as miR-34a sponge to regulate GFRA1 expression through the ceRNA mechanism. There are several lines of evidence implicating that circGFRA1 functions as a ceRNA to GFRA1 in TNBC as a sponge of miR-34a. First, bioinformatics analyses showed that the 3′UTR of both circGFRA1 and GFRA1 contain binding sites for miR-34a. Second, luciferase reporter assays verified this prediction. Third, knockdown of circGFRA1 reduced expression of GFRA1. Finally, inhibition of miR-34a reversed the effect of circGFRA1 knockdown. All the above results suggest that circGFRA1 and GFRA1 is a couple of ceRNAs that are linked by miR-34a.

## Conclusions

Taken together, our study indicates that circGFRA1 is upregulated and correlated with poor clinical outcomes in TNBC. circGFRA1 could promote proliferation and inhibit apoptosis in TNBC. And circGFRA1 functions as a ceRNA to regulate GFRA1 expression by decoying miR-34a in TNBC progression. circGFRA1 can be used as a diagnostic biomarker and potential target in TNBC therapy.

## Abbreviations

ceRNA: competing endogenous RNA
circGFRA1: circular RNA GFRA1
lncRNAs: long noncoding RNAs
qRT-PCR: quantitative real-time PCR
TNBC: triple negative breast cancer
